# Surgical Management of an *Aspergillus* Empyema in a 3-Year-Old Child

**DOI:** 10.1155/2020/5179292

**Published:** 2020-05-05

**Authors:** Meletios A. Kanakis, Konstantinos Th. Petsios, Nicholas M. Giannopoulos, Dimitrios Bobos, Sofia Hatzianastasiou, Lida C. Sianidou, Achilleas Lioulias

**Affiliations:** ^1^Department of Pediatric and Congenital Heart Surgery, Onassis Cardiac Surgery Center, Athens, Greece; ^2^Clinical Research Office, Onassis Cardiac Surgery Center, Athens, Greece; ^3^Hellenic Center for Disease Control and Prevention, Athens, Greece; ^4^Pediatric Intensive Care Unit, “PaidonPentelis” Children's Hospital, Athens, Greece; ^5^Department of Thoracic Surgery, Sismanoglio General Hospital of Athens, Athens, Greece

## Abstract

*Aspergillus* empyema in nonimmunocompromised children is rare. A case of surgical management of invasive aspergillosis in a previously healthy 3-year-old child is presented. The patient was initially admitted to a hospital with severe respiratory deterioration and clinical instability, originally attributed to sepsis. After surgical intervention and the diagnosis of invasive aspergillosis, intravenous therapy with voriconazole was initiated. During postoperative care, the patient's condition remained stable with mild functional respiratory deficits. The diagnosis and treatment of *Aspergillus* empyema remains challenging, especially in cases that the recognition of aspergillosis is delayed and urgent surgical management of the empyema is required due to rapid clinical deterioration of the patient. The early initiation, prolonged administration, and close monitoring of high-dose antifungal treatment are highly recommended.

## 1. Introduction

*Aspergillus* empyema following an *Aspergillus* infection in immunocompetent children is exceedingly rare, and its incidence remains unknown. *Aspergillus* species are ubiquitous airborne saprophytic fungi that are responsible for a variety of lung infections and diseases. *Aspergillus* pleural empyema is usually caused by *Aspergillus fumigatus* in isolation or occasionally in conjunction with polymicrobial infections. Most commonly, the fungus develops when a degree of immune suppression is present and is also related to the presence of comorbidities (cancer, immunosuppression, diabetes, transplantation, etc.) [1, 2]. However, it is reported that critically ill children during their intensive care (ICU) stay are at high risk for aspergillosis, even in the absence of immunosuppression, especially children under treatment with corticosteroids or with underlying heart or/and lung disease [3].

It is debated whether pleural aspergillosis represents an autonomous spontaneous presentation or is a complication of a deep-seated *Aspergillus* infection. In most cases, the latter seems to hold true. Since the global burden of chronic pulmonary aspergillosis is increasing, while the disease is still underreported and underdiagnosed, its late identification via complications is on the rise [2, 3]. Concerning the treatment of *Aspergillus* empyema, it seems that there is no consensus regarding the best care pathway; however, it is acknowledged that a combination of prolonged antifungal therapy, surgical resection, and chest drainage seems to bring the best outcome [1, 4].

Here, we present the rare case of a previously healthy 3-year-old child who developed *Aspergillus* empyema and presented with severe respiratory deterioration and clinical instability, originally attributed to sepsis.

## 2. Case Presentation

A 3-year-old boy was admitted to a pediatric hospital with a presumptive diagnosis of sepsis, after a two-week history of malaise and poor appetite and a single episode of haemoptysis the previous day. His past medical history was remarkable for a severe staphylococcal pneumonia, for which the child had been intubated and hospitalized for a month. He had been discharged from the hospital in a good clinical condition five months prior to his present admission, with residual large bullae in the left upper lobe. The child had been fully immunized for his age and had otherwise no history of recurrent infections or other clinical indication of immune deficiency.

**Figure 1:**
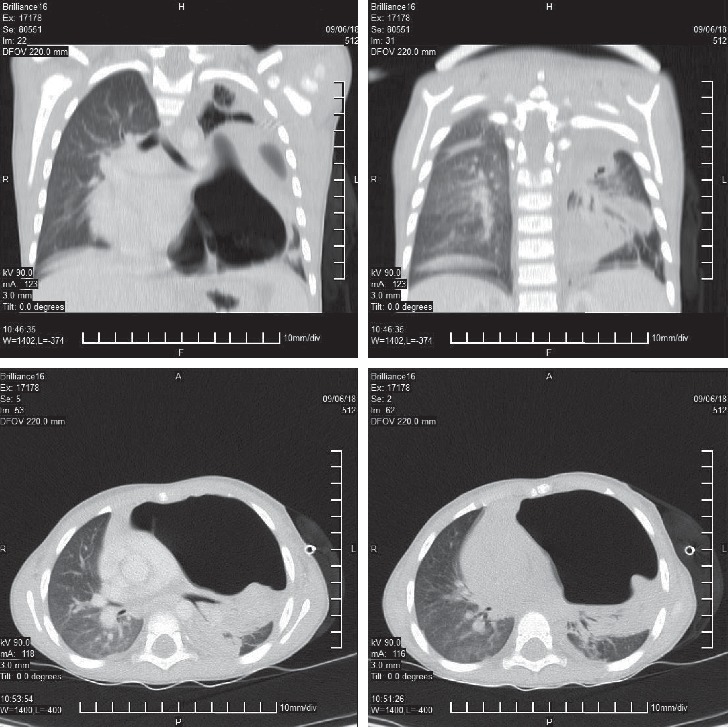
Chest CT images illustrating the extensive *Aspergillus* empyema of the left hemithorax.

During the admission to the pediatric hospital, the child clinically deteriorated and underwent endotracheal intubation. Α left chest drain was placed in order to evacuate air and fluid from the left hemithorax, as the chest CT scan was suggestive of pneumothorax and pleural effusion (Figure 1). The initial laboratory evaluation revealed a while cell count of 11.000/ml and an elevated CRP of 230 mg/L (cutoff, 5 mg/L), while the biochemical profile was normal and four sequential blood cultures eventually showed no growth.

Subsequently, the child was referred to our tertiary care hospital for surgical management. A left posterolateral thoracotomy was performed via the 5^th^ intercostal space. Intraoperative findings were consistent with extensive empyema deriving from the left upper lobe of the lung. Pleural fluid and splanchnic and parietal pleura samples were collected for cultures. Both lobes were decorticated and mobilized. Thorough haemostasis and aerostasis were performed, and the patient was transferred to the ICU in a stable condition. He was extubated on postoperative day 1.

Pleural fluid and pleura specimens cultured on a Sabouraud dextrose medium confirmed the diagnosis of *Aspergillus* empyema. A Gram stain of the empyema fluid revealed septate fungal hyphae branching at acute angles of approximately 45° (dichotomous branching), the characteristic of *Aspergillus,* which the microbiology lab orally communicated as *Aspergillus fumigatus* but subsequently reported as *Aspergillus sp* in writing. Blood cultures were negative. Given that *Aspergillus* was grown by culture of a specimen obtained by a sterile procedure from a normally sterile and clinically and radiologically abnormal site consistent with an infectious disease process, this case fulfils the criteria of a proven invasive aspergillosis [5]. Galactomannan assays and molecular diagnostic methods were not performed, as they are not available in our hospital.

A thorough history review after this unexpected finding revealed that the boy had spent a three-week convalescence period at his grandparents' home after his hospitalization for staphylococcal pneumonia 5 months earlier. This home was adjacent to a chicken farm, which we assume to have been the source of heavy *Aspergillus* colonization of the residual pulmonary bullae due to the child's previous staphylococcal pneumonia.

Antifungal therapy with intravenous voriconazole monotherapy was initiated postoperatively for 6 weeks. An initial dose of 8 mg/kg IV q12 hr was reduced to 5 mg/kg IV q12 hr, due to an elevation of transaminases (4 times above upper normal value). Although voriconazole blood level monitoring is desirable during the treatment of invasive *Aspergillus* infections, this was not available in our hospital. We therefore monitored treatment via daily clinical evaluations, serial laboratory tests twice weekly, and chest x-rays fortnightly during the child's hospital stay. Laboratory tests included a full blood count, potassium and magnesium levels, and liver function tests. Other than a residual mild elevation of transaminases (1.5 times above upper normal value), no other undesirable effect was noted. An uneventful recovery ensued, and the boy was discharged to pediatric care on an additional oral voriconazole treatment for 4 weeks (same dosage). The boy remained in an excellent clinical condition during the 1-year follow-up after the end of treatment.

## 3. Discussion

*Candida* species are the most common pathogens in fungal empyema thoracis. *Candida* empyema thoracis has been reported as a complication of operation, gastropleural fistula, and spontaneous esophageal rupture. Empyema thoracis caused by filamentous fungi is rare, and only sporadic cases have been reported. *Aspergillus* empyema thoracis is uncommon and may be caused by rupture of an aspergilloma cavity or as a complication of a preexisting chronic empyema [6].

In our case, the patient admitted to our hospital deteriorated rapidly after a single episode of haemoptysis and developed severe respiratory failure requiring mechanical ventilation and inotropic support. Therefore, urgent surgical management was preferred over medical treatment. The child's medical history was remarkable for a previous admission for staphylococcal pneumonia, six months prior to his current admission. That severe staphylococcal infection resulted in residual large pulmonary bullae, presumably heavily colonized by *Aspergillus* after his discharge from the hospital to a residential area with heavy loads of *Aspergillus* spores. Empirical antimicrobial therapy with amoxicillin was initially administered in the admitting pediatric hospital.

The diagnosis of invasive aspergillosis was made on the basis of pleural fluid microscopy (Gram stain) and culture of pleural fluid and pleural tissue on general purpose media that support the growth of fungi, including molds, specifically Sabouraud's dextrose medium. Modern diagnostic methods, such as molecular diagnostics and immunoassays, namely, the galactomannan test for *Aspergillus* antigens, are increasingly being used but are, however, not universally available in hospital settings, in which case diagnosis still relies on microscopy and culture [7]. It is to be noted that blood cultures are rarely positive in cases of proven invasive tissue aspergillosis and were indeed negative in our case.

Immediately after the identification of *Aspergillus*, intravenous voriconazole was initiated. Despite the high mortality rate, there is no established standard antifungal therapy for *Aspergillus* empyema. The IV voriconazole formulation is widely recommended in seriously ill patients since it has been related to better survival and lower incidence of adverse effects. The currently recommended dose of voriconazole in pediatric patients over 12 years of age is 6 mg/kg IV for the first 24 hours, then 4 mg/kg IV q12 hr [4]. For children aged 2–12 years and adolescents with low body weight (<50 kg), the recommended dose of voriconazole is 9 mg/kg IV for the first 24 hours, then 8 mg/kg IV q12 hr. In addition, the Infectious Diseases Society of America recently recommended drug monitoring in all pediatric patients weighing less than 50 kg and noted that higher than standard voriconazole doses might be needed, since it is common for pediatric patients to present increased pharmacokinetic variability [4, 8].

In our case, we administered IV therapy for 6 weeks (5 mg/kgr/12 h) followed by an additional 4 weeks of oral intake (same dosage). The initial higher dosage of 8 mg/kgr/12 h was poorly tolerated due to deranged liver function. The duration of antifungal treatment was guided by clinical response and inflammation markers, since there are no established guidelines in pediatric or adult patients. Therapeutic drug monitoring was unfortunately not available in our hospital. We therefore followed an approach of close clinical and laboratory monitoring for treatment efficiency, based on respiratory function indices, serum inflammatory markers, and serial chest X-rays. Liver function tests were monitored as a marker of eventual toxicity.

Burgos et al. [9] found no difference among the different antifungal regiments in their multivariate analysis. Interestingly, they reported that the only predictor of survival was surgical resection of focal disease. On the other hand, Burgos et al. claim that superior outcomes in surgically treated patients can be attributed to an overall better immune status and more stable clinical condition rather than to the surgical intervention itself.

In our case, the combination of medical and surgical approach proved beneficial for the patient. Surgical intervention is mostly manageable with localized disease and clinical stability. We, however, decided to proceed to an urgent surgical intervention, even though the patient was severely compromised clinically, by evaluating the advantage of such an approach for the survival and the respiratory function of the child.

Multiple surgical techniques have been employed to achieve better functional respiratory outcomes including removal of infected chest wall implants used to reconstruct either the chest wall, diaphragm, or pericardial surfaces after pneumonectomy [10], thoracic window techniques to allow drainage with subsequent spongostan and amphotericin B talc poudrage [11], surgical debridement of only the bronchial stump following lobectomy/pneumonectomy [1], pleuro-pneumonectomy and nystatin irrigation [12], thoracotomy and decortication of the affected side [13, 14], and thoracoplasty [15].

More complicated procedures include pneumonectomy with thoracoplasty to obliterate the pleural space and provide tissue coverage for the bronchial stump [16]; thoracotomy, pleurectomy, and placement of a muscle flap for limited disease [17]; thoracomyoplasty being employed for more extensive disease [17]; thoracostomy with daily insertion of gauze impregnated with amphotericin B followed by either a muscle [18] or an omental flap [17, 19], and the Eloesser procedure [20]. The Eloesser procedure is less disfiguring than a free muscle flap or extensive thoracoplasty, while it still supports the creation a deep pleurocutaneous fistula, allowing drainage of the pleural cavity whilst maintaining negative pressure through the creation of a one way valve. The inner opening of the flap should be sealed as the lung expands with increased drainage of pleural fluid.

More recently, video-assisted thoracoscopy has been reported as being successful in the treatment of an *Aspergillus* empyema following failure of medical therapy, with complete resolution and no recurrence at five months [21].

Despite the wealth of techniques available, complications following surgery of any kind can be severe and include necrosis of the muscle flap, haemorrhage from the bronchial stump, persistent air leak, and multiorgan failure due persistent infection [16, 17].

In our case, after the surgical operation, our patient was stabilized in the pediatric intensive care unit for 7 days. The patient's condition was stable throughout with mild respiratory deficits. During his ICU stay, the patient had to be closely monitored for lung expansion and respiratory function, as well as for the lack of active bleeding in the chest drainage, fever, and negative fluid balance.

## 4. Conclusion

The treatment of *Aspergillus* empyema is challenging, particularly in cases when the diagnosis of invasive aspergillosis is delayed and urgent surgical intervention is required due to rapid clinical deterioration. The early initiation, prolonged administration, and close monitoring of antifungal therapy are highly recommended. In most cases, monotherapy with voriconazole is the treatment of choice, except in cases when a combination of antifungal agents is deemed necessary according to the antifungal drug sensitivities or in cases that voriconazole is not effective. In addition, higher dosages of antifungal agents may be administered to young children in comparison to older children and adults in order to ensure a satisfactory clinical response.
